# Delayed subsidence of the Dead Sea shore due to hydro-meteorological changes

**DOI:** 10.1038/s41598-021-91949-y

**Published:** 2021-06-29

**Authors:** Sibylle Vey, D. Al-Halbouni, M. Haghshenas Haghighi, F. Alshawaf, J. Vüllers, A. Güntner, G. Dick, M. Ramatschi, P. Teatini, J. Wickert, M. Weber

**Affiliations:** 1 Deutsches GeoForschungsZentrum, GFZ, Telegrafenberg, 14473 Potsdam, Germany; 2 GEOMAR - Helmholtz Centre for Ocean Research, Kiel, Germany; 3 Leibniz University Hanover, Hanover, Germany; 4 Karlsruhe Institute of Technology, Karlsruhe, Germany; 5 University of Potsdam, Potsdam, Germany; 6 University of Padova, Padua, Italy; 7 Technische Universität Berlin, Berlin, Germany

**Keywords:** Hydrology, Geophysics

## Abstract

Many studies show the sensitivity of our environment to manmade changes, especially the anthropogenic impact on atmospheric and hydrological processes. The effect on Solid Earth processes such as subsidence is less straightforward. Subsidence is usually slow and relates to the interplay of complex hydro-mechanical processes, thus making relations to atmospheric changes difficult to observe. In the Dead Sea (DS) region, however, climatic forcing is strong and over-use of fresh water is massive. An observation period of 3 years was thus sufficient to link the high evaporation (97 cm/year) and the subsequent drop of the Dead Sea lake level (− 110 cm/year), with high subsidence rates of the Earth’s surface (− 15 cm/year). Applying innovative Global Navigation Satellite System (GNSS) techniques, we are able to resolve this subsidence of the “Solid Earth” even on a monthly basis and show that it behaves synchronous to atmospheric and hydrological changes with a time lag of two months. We show that the amplitude and fluctuation period of ground deformation is related to poro-elastic hydro-mechanical soil response to lake level changes. This provides, to our knowledge, a first direct link between shore subsidence, lake-level drop and evaporation.

## Motivation

The Dead Sea is a hyper-saline terminal lake located in the Dead Sea transform rift system^1–3^. Today its catchment area provides fresh water for more than 16 million people in Jordan, Israel and the Palestinian territories^4^. In recent decades, this region has faced substantial environmental challenges^5^; water scarcity is one of the most serious. Since the 1950s, anthropogenic influence led to an unprecedented recession of the Dead Sea^4–6^. The lake level has steadily decreased with now more than − 110 cm/year^7^, leaving the level in October 2018 at 433 m below mean sea-level (msl).

There are several reasons for the net loss of lake volume in the water balance of the Dead Sea^5,8^: (1) A high net evaporation rate of around 1000 mm/year (~ 700 × 10e6 m^3^/year) with large seasonal variations^9^ of which the quantification has recently been improved by new eddy covariance measurements^10^; (2) Extensive use of the DS brine for Potash production in Israel and Jordan with a net water usage in the order of 250 × 10^6^ m^3^/year is estimated to be responsible for 40% of the lake level drop^8,11^; (3) Large water irrigation projects in the North^12^, causing fresh water inflow of the Jordan River to decrease by 90% compared to the natural situation before 1955, to 60–400 × 10e6 m^3^/year nowadays^11,13,14^.

Generally, surface and subsurface water inflow into the Dead Sea are difficult to determine due to complex geology and spatio-temporal effects^8,15–17^. Regional-scale 3D hydro(geo)logical modelling in salt-water environments has shown the most promising results for the Dead Sea aquifer systems^18–21^. Water inflow into the DS comprises direct surface runoff from river basins with a volume of 58–66 × 10^6^ m^3^/year (excluding the Jordan River), submarine groundwater discharge for the Lower Cretaceous Aquifers of ca. 170 × 10e6 m^3^/year and precipitation on the lake surface of ca. 45 × 10^6^ m^3^/year^8,11^. These inflows, which additionally tend to decrease due to climate change^21–23^ cannot compensate for the high evaporation.

The rapid decline of the DS level leads to both short and medium term climatic changes and natural hazards^5,24,25^ that pose a major challenge to local communities^26^. Changes in precipitation and evaporation cause major flooding events, desertification and land degradation^4,26,27^. The retreat of the salt-water to fresh-water transition zone at the DS shore^28^ results in an increasing groundwater gradient^7,29^. Both developments have led to dissolution and erosion processes of the DS sediments, evaporates (salt) and other soluble material on both sides of the Dead Sea^30–32^. As a consequence, strong subsidence in the order of mm to cm/month on different spatial scales^7,33–35^ and hazardous local sinkhole phenomena occur^30,31,36^, with disastrous effects on infrastructure, industry, tourism and agriculture^6,37,38^.

Determining the link between land subsidence and lake level change is essential for understanding the physical processes behind. Subsidence estimation at the Dead Sea has been performed locally by close-range photogrammetry, interferometric synthetic aperture radar (InSAR) or LiDAR techniques or by reconstruction of the Dead Sea bathymetry^7,33,34,36,39–43^. In contrast, regional studies exist that yield contrasting results, interpreted as lithostatic rebound^46^.

To shed light on the link between evaporation rate, lake-level decline and ground subsidence we use a recently acquired and compiled dataset in the framework of the interdisciplinary DESERVE (DEad SEa Research VEnue) project^5^. Ground deformation is hereby measured with up-to-date high precision Global Navigation Satellite System (GNSS) stations. GNSS reflectometry is used for high precision leveling. GNSS is especially suitable for monitoring high temporal land subsidence variations as expected in natural and man-made (mining) cases e.g. Refs.^45–48^. We use this technique to record the temporal subsidence at the western side of the Dead Sea, in different distances to the shore line. We compare, in high temporal resolution, evaporation and lake level changes at the Dead Sea and are able to determine a first direct and interdisciplinary link on a monthly basis between the Solid Earth, climate and water processes.

## Study area and experiment description

**Figure 1:**
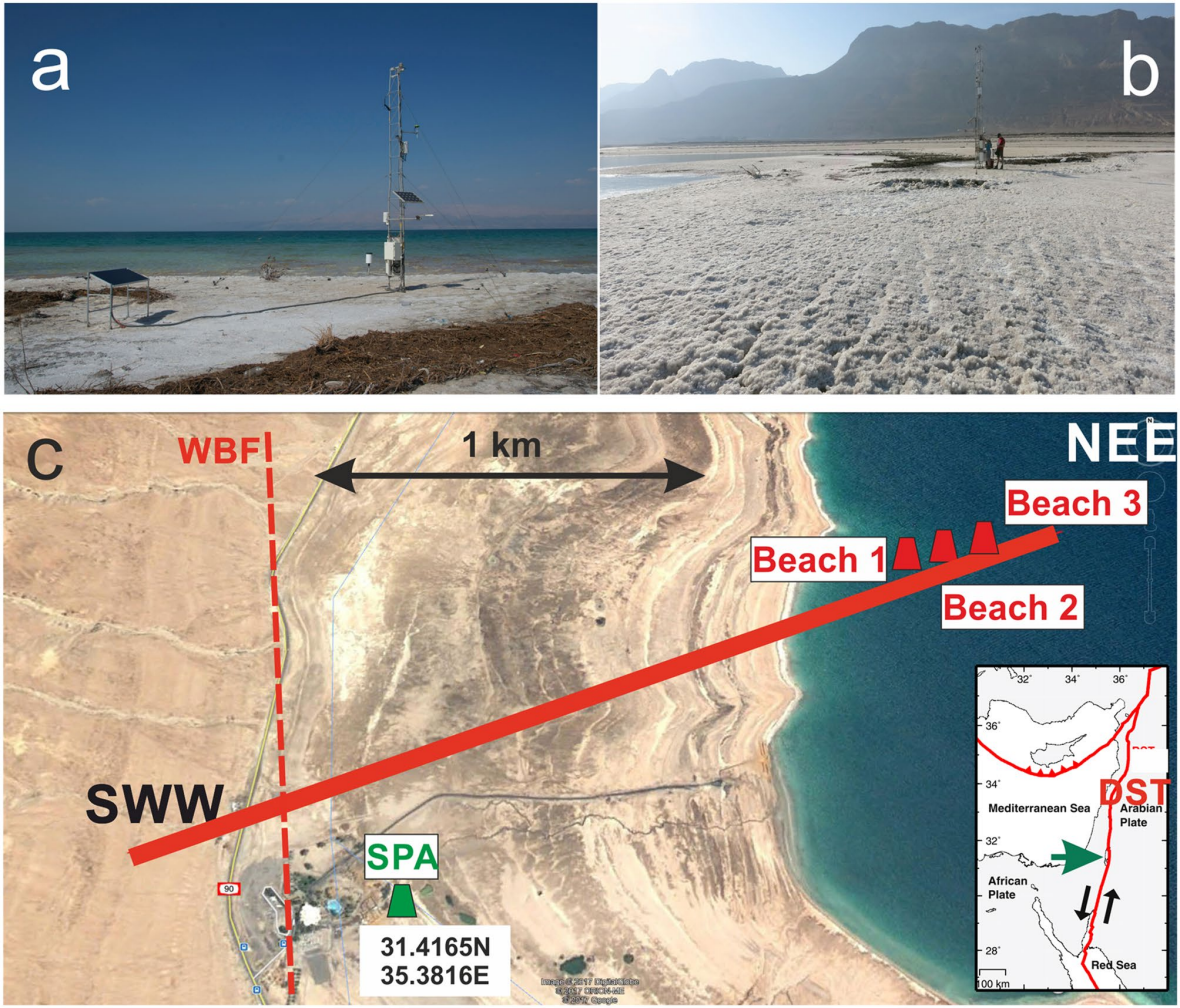
(**a**) GNSS station in June 2014 (Beach 1). (**b**) GNSS station in December 2014 (Beach 1). (**c**) Study area at the Dead Sea (DS) near Ein Gedi, Israel, with the four GNSS observation sites as green (SPA, with coordinates) and red (Beach) symbols, respectively. The solid red line shows the profile in Fig. 5 (2.4 km length). Note that the Google image is from 2010. In 2014, the DS had sufficiently receded so that the Beach 1 station was on land. This station had then to be moved twice to be near the lakeshore. *WBF* Western Boundary Fault (dashed line), a fault of the Dead Sea Transform (DST) system. (Insert) Study area at the DS (green arrow). Black arrows indicate the left lateral displacement of 105 km at the DST^27^. Image © 2017 Digital Globe, © 2017 ORION-ME, © 2017 Google.

Figure 1 shows the tectonic setting of the DS and the location of the Global Navigation Satellite System (GNSS) stations used. The study area is located on the west coast of the DS near Ein Gedi, Israel, at 31.41° N, 35.39° E. Meteorological and GNSS data were recorded for nearly 3 years from 11th June 2014 until 29th March 2017. The SPA site had a fixed location next to the spa area of Ein Gedi. As the coastline of the DS retreats about 100 m/year horizontally, the Beach site had to be moved twice towards the waterfront in spring 2015 and 2016, respectively. The GNSS antennas are mounted about 4 m above the ground on towers with meteorological sensors (tipping bucket rain gauge 52,202 from Young IRGASON; integrated CO_2_/H_2_O open-path gas analyzer and 3D sonic anemometer from Campbell Scientific) installed by the Karlsruhe Institute of Technology (KIT) in the framework of the DESERVE project^5^. The GNSS equipment consists of an OEM receiver board type Javad TRE_G3T and an antenna Javad JAV_GRANT-G3T without radome. The ground around the Beach station is a slightly undulating, massive and rock-hard salt crust of 10–20 cm thickness (Fig. 1b). Below this stable lid of salt, the soft clayey sediments are brine-/water-logged. GNSS-reflectometry, a method that uses the GNSS signals reflected from land, snow and water surfaces is applied to monitor the Dead Sea lake level change^49–54^. For details on the data analysis and the construction of 3-year GNSS time series, the reader is referred to the supplement and Supplementary Figs. S_1 and S_2, respectively.

## Observations

**Figure 2:**
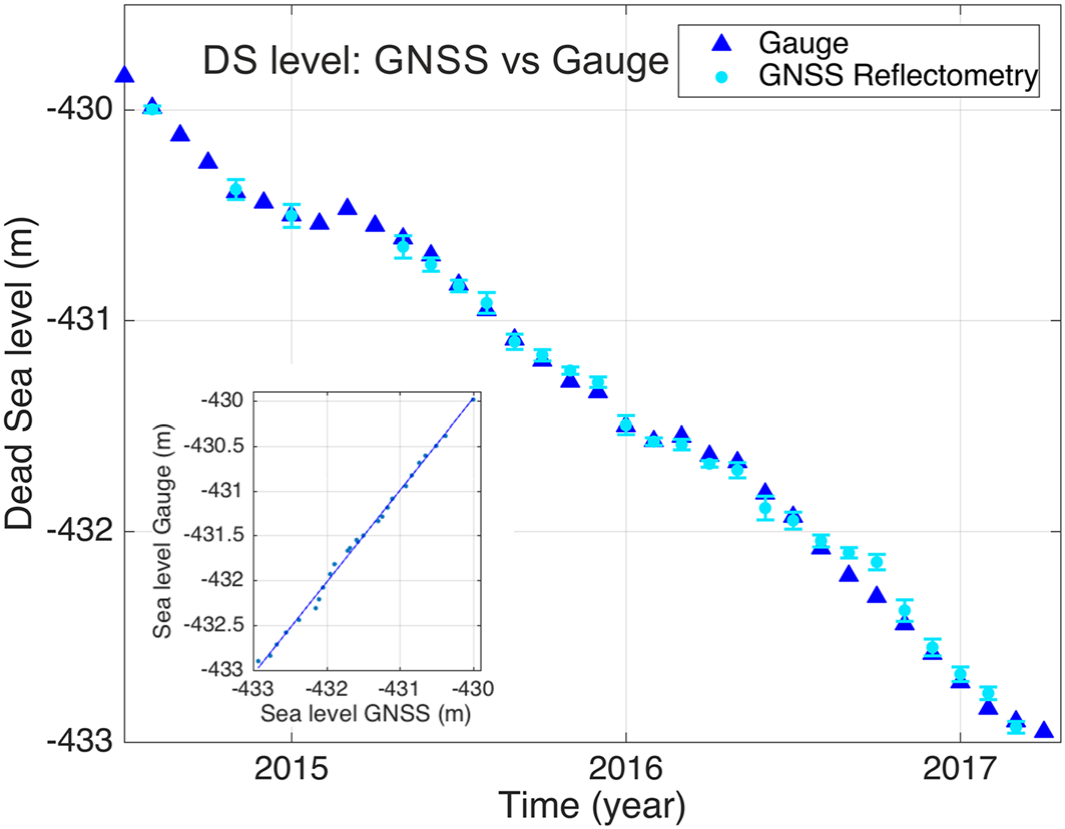
Blue triangles represent the DS lake level from 11th June 2014 to 29th March 2017, in meters below mean sea level, data provided by the Hydrological Service and Water Authority, Israel (Gauge near Massada). The DS lake level determined from GNSS reflectometry observations (Fig. 1, Beach stations) is shown in light blue (dots), with error bars. The standard error of the GNSS measurements is ± 2.7 cm. (Insert) The correlation coefficient between the two time series is 0.99, indicating the high accuracy of the GNSS observations.

To validate the GNSS reflectometry method, we compare the GNSS derived DS lake level with gauge measurements near Massada (31.32863 N, 35.40299 E) from the Hydrological Service and Water Authority in Israel, for the 3-year observation period (Fig. 2). The mean deviation between the DS lake level from gauge observations and the lake level derived from GNSS reflectometry is ± 2.7 cm. The correlation coefficient of 0.99 ± 0.001 indicates an excellent accuracy and robustness of the GNSS method. The linear trend for the 3-year observation period (2014–2017) of the DS lake is − 110 ± 7 cm/year.

**Figure 3:**
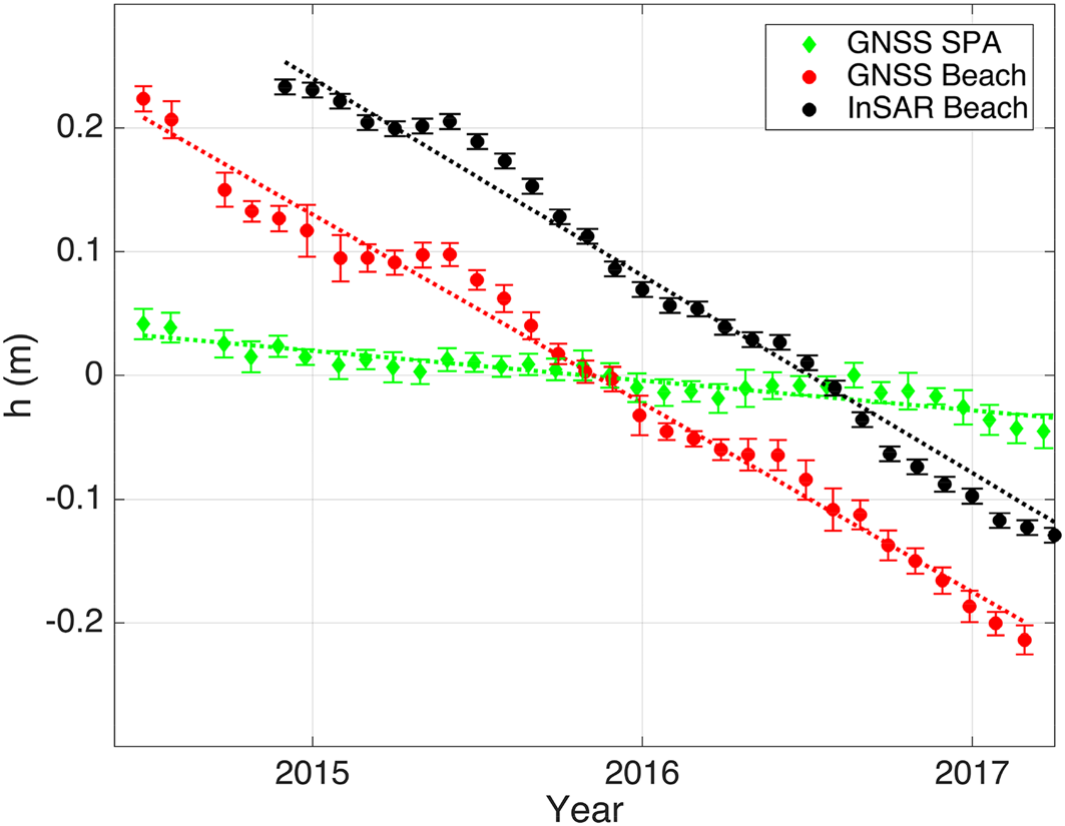
Displacement relative to the middle of the observation period is shown for the GNSS station at the SPA (green symbols) and the Beach stations (red symbols), respectively, between June 2014 and March 2017, in meters. The average subsidence at the SPA is − 2.4 ± 0.7 cm/year that at the beach is − 15.3 ± 1.2 cm/year, respectively. The standard error of the monthly mean GNSS height measurements is ± 1.1 cm. The subsidence at the beach derived from InSAR is − 15.9 ± 1.5 cm/year.

Figure 3 shows the vertical movement of the land surface at the SPA and Beach stations for the whole observation period. The standard error per month is ± 1.1 cm and the average subsidence is − 2.4 ± 0.7 cm/year and − 15.3 ± 1.2 cm/year at the SPA and Beach stations, respectively. The subsidence at the Beach station derived from InSAR corresponds with − 15.9 ± 1.5 cm/year very well to the GNSS observations. The spatial distribution of the subsidence between the Beach and the SPA stations is shown in the supplementary material for InSAR images (Supplementary Fig. S_4).

## Seasonal variation of subsidence

**Figure 4:**
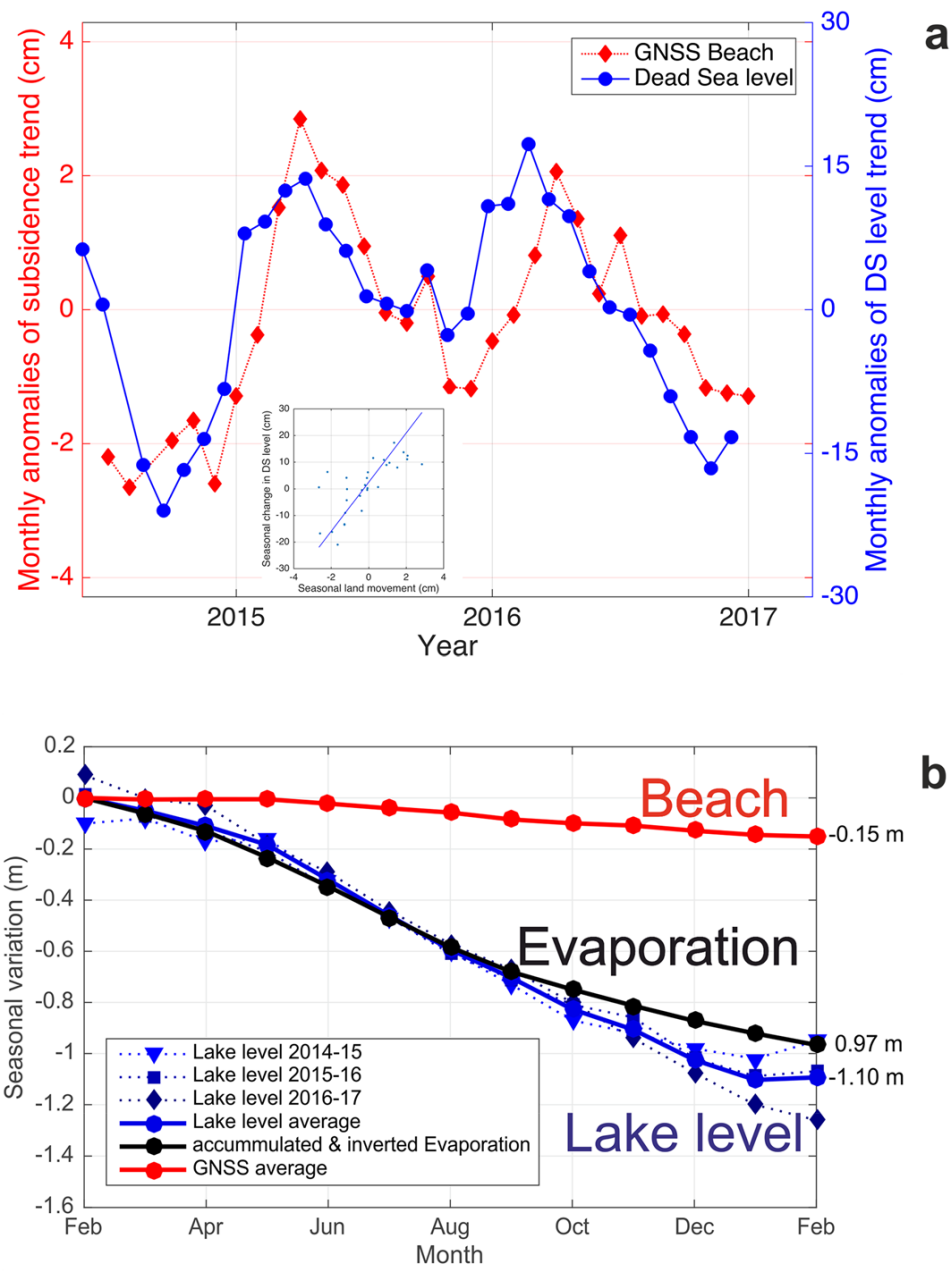
(**a**) Monthly anomalies of the subsidence of the Beach station (red, from GNSS) and of the drop of the lake level of the DS (blue, gauge) from June 2014 to March 2017, after removal of their respective 3-year trends (− 15.3 cm/year and − 110 cm/year). The standard error for the subsidence anomalies is ± 1.1 cm and for the lake level anomalies is ± 2.7 cm. (Insert) Correlation between the two time series after backward shifting of the subsidence time series by 2 months, with a correlation coefficient of 0.84 (see also Supplementary Fig. S_3). (**b**) Absolute values for Beach subsidence (red), lake level drop (blue) and evaporation (black), respectively, for three 12-month periods from February to February (2014/2015, 2015/2016, 2016/2017). The solid lines depict the respective average over the three years; the dashed lines are the values for the single years. The winter 2014/2015 was wet (see Supplementary Fig. S_6) and that of 2016/2017 was dry, both visible in higher or lower lake level values, respectively. The values for the cumulative evaporation are based on Ref.^10^. The scaling factor between Beach (land) and the DS lake level is 7.2; i.e. − 100 cm DS lake level decrease corresponds to − 13.9 cm sinking of the beach.

To isolate the seasonal signal of the lake level drop and of beach subsidence from the general trend we remove the 3-year trend from the data. The Beach station shows a seasonal signal in the subsidence while the SPA station does not (Fig. 4). Positive anomalies of the overall decreasing DS lake level trend (positive numbers in Fig. 4a) are related to precipitation and less evaporation in the winter months. Anthropogenic water usage also plays a role and is discussed below. The main subsidence at the Beach site occurs from May to January (given as an anomaly of − 1.3 cm/month relative to the long-term subsidence in Fig. 4a), whereas it is significantly smaller from January to May (anomaly of 0.5 cm/month). The subsidence at the beach shows the highest correlation (0.84) with the DS lake level for a delay of two months (see Supplementary Fig. S_3), see also insert in Fig. 4a.

Figure 4b shows the subsidence of the beach, the lake level and the accumulated evaporation determined from data recorded at the same meteorological tower on which the GNSS antenna was mounted^10^. For details see supplement and Supplementary Fig. S_3. High correlations between evaporation/lake level and subsidence are expected since the fine-grained sediments cause complete consolidation quickly^33,55^. The scaling factor between absolute lake level change and subsidence is 7.2 (Fig. 4b). This means that the beach drops—with a time delay of 2 months—with the lake level, but at a 7.2 times smaller rate.

The lake level drop (− 110 cm/year) is larger than the evaporation observed at this location (97 cm/year). Firstly, this is due to the fact that the inflow of the Jordan river is massively reduced through overuse for consumption and that secondly the brines of the DS are used by industry ~ 30 km south of our observation point^5,8,11^. The sum of evaporation losses and of water withdrawals for the potash production, which amount to close to half of the evaporation losses, cannot be compensated by the surface and subsurface inflow to the DS and cause the long-term decline of its water table^3^.

## Discussion

### Origin of land subsidence

Land subsidence at the Dead Sea region occurs on different spatial and temporal scales^7^:

1. Meter to decimeter scale sinkholes are related to subsurface material dissolution and mechanical mobilization^30,32^ either due to dissolution of a salt edge^56,57^, structurally controlled groundwater percolation^58,59^ or subsurface stream channels^36,60,61^ Formation rates vary hereby from sudden (e.g. within seconds) or in the order of mm-cm/month as determined by photogrammetric/InSAR/LiDAR studies^34,36,40,41,44^ and morphologies of such sinkholes vary according to mechanical properties of the overburden^66,67^.
2. Hundred meter scale depressions as common karstic landforms, so called uvalas^62^, usually with sinkhole formation in parallel or earlier than the depressions with a strong temporal relation to base-level fall and structural trends as well as the fresh-salt-water boundary^7,58,59^.
3. A large-scale distributed non-linear subsidence with rates between 0.01 and 0.3 m/year depending on the distance towards the shoreline^7^. The rates agree well with previous InSAR or photogrammetric studies^33,41,43^, including the results presented in this study. Effects of subsurface channel or local dissolution related subsidence close to or above active channels can be ruled out for the study site discussed here, as we observe rather opposite patterns of seasonal subsidence variation compared to^42^. The nearest visible surface channel is in a distance of ~ 750 m and the nearest sinkholes are reported to occur several hundreds of meters to the north and west of the Beach station^35,63^, and also a few hundred meters from the SPA station. Given the typical size distribution of sinkholes in clayey marl/alluvial sediments^36,40^, ground subsidence due to sinkholes would not be observed in the footprint area (Fresnel zones) of the GNSS antenna for our study sites (see Supplementary Information). Also, uvala formation in either cover material is usually accompanied by large-scale crack formation^7^, something not observed for the area close to the GNSS stations.

### Soil mechanics considerations

The formation of subsidence generally depends also on the rock/soil mechanical properties, as highlighted in various numerical modelling studies from Refs.^39,64–67^. A drop in pore pressure due to the decline of the lake level and, thus, in the fine-grained sediments along the shoreline, causes the sediments to consolidate^33^. The main subsurface material in the area is clayey marl and dewatering is a complex process involving kinetic, thermodynamic and electrochemical aspects^68^. Broad-scale subsidence along the DS shoreline has been attributed to such compaction of fine-grained formerly water-logged sediments, and was estimated to several cm/year for marl deposits of the study area by analytical considerations^33^. The sediments of the Dead Sea, alluvium, clayey marl deposits and salt, have distinguished mechanical strengths and behavior ranging from brittle- to ductile failure^53,64,68,69^, and salt concentration of water-clogged sediments has a significant influence on shear strength and Atterberg limits^70^. In combination with the above-mentioned missing evidence of sinkhole and uvala formation, we therefore consider option (3) from above, the large-scale compaction of the former Dead Sea lake-bed, as the most probable process that causes the observed subsidence.

We present analytical calculations of ground subsidence and water level fluctuation propagation by applying simple analytical 1D-soil compaction theory based on Refs.^71,72^. This assumes, for simplicity, a 20 m thick unconfined, isotropic, homogeneous and fully saturated Dead Sea brine layer of marl overlying a thick Holocene salt layer. For a water-level decrease of 1.1 m (corresponding to the mean annual Dead Sea water level decline, see Fig. 4b), the results show a 1D solid consolidation of 10.2–17.0 cm with a primary consolidation time of 1.2–3.6 years. The observed values at the station Beach are within this range. Further details on the analysis and assumptions made can be found in the supplement.

Delayed groundwater pressure propagation may be the primary reason for the observed time shift between DS lake-level drop and subsidence. In fact, assuming the aquifer system as a poro-elastic body, a seasonality of the subsurface fluid pressure causes a simultaneous behavior of soil deformation because of the effective stress fluctuation^71,73^. To test this hypothesis we used the 1D analytical poro-elastic response of an aquifer system subject to lake-level fluctuations. Specifically, the interest is to understand how the signal represented by the DS level after removal of the 3-year trend propagates inside the beach from the DS. Figure 4a suggests that this residual sea level fluctuation can be viewed as a long-term tide characterized by an amplitude of ca. ${\zeta }_{0}=15$ cm and a period ${t}_{0}=365$ days. We consider a homogeneous beach of a water saturated clayey marl characterized by an isotropic permeability, constant effective porosity, horizontal groundwater flow and hydrostatic conditions to solve the governing equations for effective stress calculation based on poro-elasticity (see supplement). The distance of the Beach station to the water ranged from 10 to 35 m during the survey time. Using these values yields a time-lag of level drop propagation between ${t}_{L}=29$ days and ${t}_{L}=101$ days, respectively, which encompass the estimated time shift between the observed DS level and land movement at Beach station (ca. 60 days). Taking advantage of the previous soil mechanical computation, we can use the ratio between the yearly DS level change and the shore land displacement, i.e. $r=$ 7.2, to make a rough estimate of the possible seasonal movement above the Beach station due to the inland propagation of the seasonal sea level fluctuation. We obtain a value equal to 1.25 cm for 10 m distance from the shoreline, and 1.98 cm for 1 m distance, close to the average value ~ 2 cm, as shown in Fig. 4a. At more than 35 m distance from the shoreline the seasonal displacement becomes negligible. With the background of the fast regression of the shoreline and related exposure of the Dead Sea clayey marl, theoretical and measured values are in good agreement.

This cause-and-effect behavior has been already observed above underground gas storage reservoirs^74^, alluvial confined aquifers cyclically exploited^75^, and fractured rock aquifers experiencing Earth tides^76^. Recently, albeit for different hydrogeological settings, a time lag of about 45 days between surface displacements and variations of the groundwater level was published^77^, in line with the observations obtained here. However, to our knowledge, it is the first time that this has been directly recorded on a shore.

We would like to point out, however, that variations of compression indices and coefficients of consolidation with time, Atterberg limits and pre-consolidation pressure were not considered in the consolidation calculations. Also, 3D effects and non-homogeneous soils, e.g., with respect to their lime content^78^, may play a role and have not been considered here. The general lack of similar high-frequency subsidence data prevents us from assessing our results in a broader context. Spatially varying layer depths, material heterogeneity and liquefaction, and the groundwater-fresh water interface locally penetrated by subsurface conduits are responsible for the different magnitudes of large-scale subsidence along the Dead Sea shoreline^7^ and also for a location-dependent time lag between water level drop and subsidence. For analyzing these effects more accurately, longer high-resolution measurements on several locations along the Dead Sea would be necessary. Nevertheless, the values observed at the Beach site show a valuable first benchmark for the rate of subsidence by poro-elastic consolidation of silt and clay sediments along the DS shoreline, which is highly uncertain due to the difficulties to determine sediment properties^55^.

### Hydrogeological and tectonic considerations

**Figure 5:**
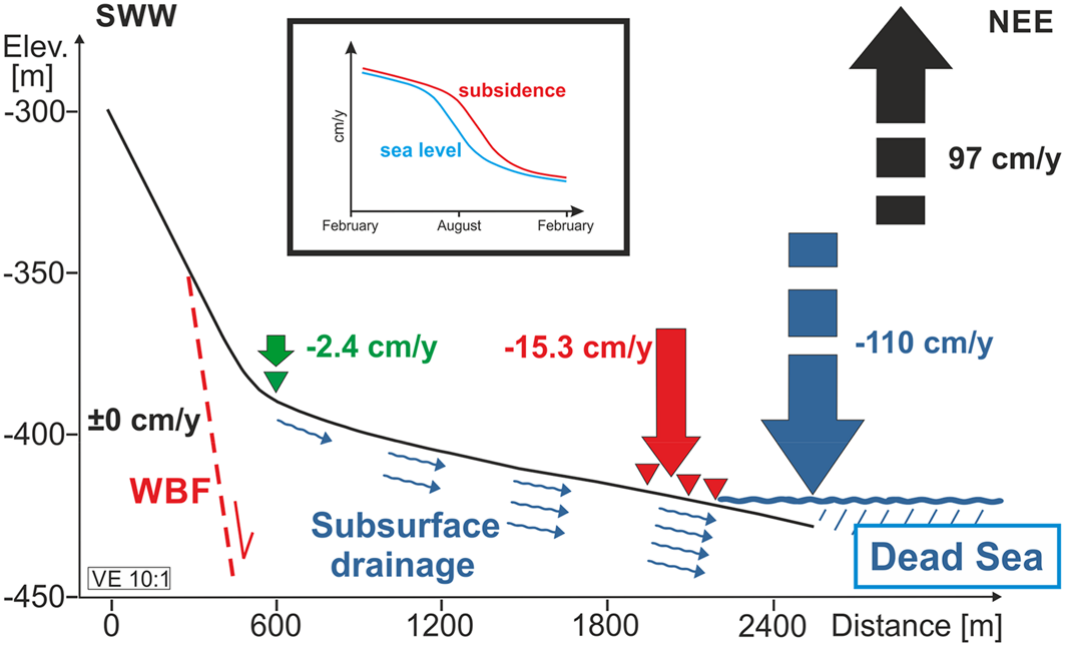
Sketch of the average yearly subsidence across the red profile shown in Fig. 1, from June 2014 to March 2017. The green and red triangles are the location of the GNSS stations. Green and red arrows indicate the subsidence of the surface. The area west of the WBF (Western Boundary Fault, dashed red line) shows no vertical movement (see Supplementary Figs. S_4 and S_5), whereas the subsidence increases from − 2.4 cm/year near the SPA to − 15.3 cm/year at the Beach. The main cause of the massive subsidence observed is the drainage of the sediments below the stable salt crust, due to the drop of the DS water table. Water and its movement are labeled in blue. The average drop of the DS lake level is 110 cm/year. Vertical exaggeration is 10:1. The insert is a sketch showing in a generalized way the intra-annual dynamics of DS lake level (blue) and the delayed subsidence (red) (winter to summer to winter).

The high correlation (0.99) between the average annual beach subsidence (red in Fig. 4a) and lake level drop (blue) and the cumulative evaporation (black; correlation coefficient − 0.97), respectively, shows the close connection of the processes in these three spheres. Away from the shore (ca. 2 km west), the subsidence at the SPA station (− 2.4 cm/year) is much smaller (Fig. 5, green symbols). This small subsidence rate could be explained by the overlay of a residual hydrology-induced subsidence (the beach receded from this location more than 35 years ago, but the groundwater table still declines in the deeper subsurface, compaction goes on and, thus, subsidence can be expected to continue at small rates) and the tectonically driven subsidence of the DS basin of up to 0.3 mm/year^79^. Furthermore, the decline of the groundwater table as a consequence of receding lake levels tends to decrease with the distance from the shoreline^31,80^. The associated compaction of fine sediments and, thus, subsidence may be more pronounced close to the shoreline.

A regional process that affects the hydrology at the western shores of the DS is the varying groundwater inflow from the recharge areas in the mountains towards the west^31^. Due to long travel and residence times in the aquifer from the recharge areas to the DS, the seasonality of rainfall and recharge is fully dampened out in the inflow to the DS area^31^. Thus, an impact of regional groundwater flow on the seasonality of lake level and subsidence observed in this study can most likely be ruled out.

West of the Western Boundary Fault (WBF), see Fig. 1, no vertical displacement is detectable (see Supplement Information and Supplementary Figs. S_4 and S_5). The vertical subsidence observed at the beach and its seasonal dynamics are clearly dominated by local hydrological processes in the sediments, which are in turn shaped by meteorological phenomena, like the wind systems dominating the evaporation processes; for details on the wind systems see Ref.^10^.

Figure 5 summarizes the different phenomena (subsidence, lake level decline and evaporation). While the reaction chain of evaporation, lake level drop and beach subsidence is now established, the surprise is the high synchronicity—with a time lag of 2 months for the beach—of these observations, dominated by a seasonal cycle.

## Summary

The study presented here shows that the use of geophysical observation methods like GNSS reflectometry in combination with traditional techniques enables us to detect the close linkage of land subsidence with changes in lake level and climate factors such as evaporation. We demonstrate, to our knowledge, for the first time the direct link of atmospheric phenomena to Solid Earth processes using the common factor water and resolve the interplay of the spheres on a seasonal, respectively monthly basis. These dynamic processes, shaping the surface of our Earth, show the high vulnerability of our environment to complex chains of processes in the geo-sphere strongly driven by human activities.

## Supplementary Information

Supplementary Information.
