# Supplementary material for: Delayed subsidence of the Dead Sea shore due to hydro-meteorological changes
Source: Sci Rep. 2021 Jun 29;11:13518. doi: 10.1038/s41598-021-91949-y (PMC8242068; doi:10.1038/s41598-021-91949-y)
Supplement: Supplementary file 1 — Supplementary Information. [file 41598_2021_91949_MOESM1_ESM.docx]

**Electronic supplement**

**Delayed subsidence of the Dead Sea shore due to hydro-meteorological changes**

S. Vey^+, 1^, D. Al-Halbouni ^1,2^, M. Haghshenas Haghighi ^3^, F. Alshawaf ^1^, J. Vüllers ^4^, A. Güntner ^1,5^, G. Dick ^1^, M. Ramatschi ^1^, P. Teatini ^6^, J. Wickert ^1,7^, M. Weber^1, 5^

+Corresponding author, e-mail: sibylle.vey@gfz-potsdam.de

^1^ Deutsches GeoForschungsZentrum, Potsdam, Germany

^2^ GEOMAR – Helmholtz Centre for Ocean Research Kiel, Germany

^3^ Leibniz University Hannover, Germany

^4^ Karlsruhe Institute of Technology, Karlsruhe, Germany

^5^ University of Potsdam, Germany

^6^ University of Padova, Italy

^7^ Technische Universität Berlin, Germany

Sibylle Vey

GFZ

Telegrafenberg

14473 Potsdam

Germany

**GNSS analysis**

**a) Positioning**

The GNSS data analysis for positioning has been performed with the GFZ Earth Parameter and Orbit determination System (EPOS) software ^1, 2^ which is based on a least squares adjustment of un-differenced phase measurements and adheres to IERS Conventions ^3^. A linear combination of the L1 and L2 GNSS observations are used in EPOS software to eliminate the first-order ionospheric effects. For estimation of station coordinates we used the Precise Point Positioning (PPP) strategy ^4^. The main idea of the PPP strategy is the processing of each site separately, fixing the high quality GNSS orbits and clocks. Thus, the GNSS data processing splits into two steps. The first processing step includes an estimation of high accurate GNSS orbits and clocks from a global GNSS network. In this step we used about 100 globally distributed stations of the International GNSS Service IGS. Official products of the GFZ IGS Analysis Centre such as Earth Orientation parameters, GNSS orbits and clocks, have been taken as initials. We used GNSS data with a sampling rate of 2.5 minutes in 24-hour data windows and applied a cut-off elevation angle of 7 degrees. As reference frame the International Terrestrial Reference Frame ITRF2014 has been used. In the second step – PPP analysis – we performed daily estimation of GNSS station coordinates using fixed orbits and clocks of GNSS satellites from the first step. The main advantage of this strategy is the possibility of investigations of site-dependent effects, which was very important in the case of Dead Sea.

**b) Reflectometry**

The DS lake level (*DSL*) is calculated using the GNSS antenna height *H* from positioning and the height of the GNSS antenna above the reflecting surface *h_refl*

DSL=H-h_refl (1).

The reflector height of the GNSS antenna can be derived from the GNSS signal strength ^5, 6, 7, 8^. The GNSS signal power is measured as signal-to-noise ratio (SNR) and recorded as standard observation in the Receiver Independent Exchange Format (RINEX) files ^9^. The SNR from the Javad receiver has a precision of 0.05 dB. The GNSS data have a sampling rate of 1 s. The reflector height can be estimated from the interference pattern of the signal to noise ratio. By subtracting a second order polynomial from the SNR data the interference pattern was isolated. The amplitude of the SNR, given in logarithmic dB-Hz units, was converted into the linear volts/volts. The SNR interference pattern is a function of the satellite elevation angle $E$ and shows a periodic signature. Assuming a locally planar and horizontal surface the frequency f of the multipath pattern is constant when $sin(E)$ is used as independent variable ^10^. The frequency $f=\frac{4\pi h}{\lambda}$ of the interference pattern depends on the height $h$ of the GNSS antenna above the reflecting surface and on the wavelength $\lambda$ of the GNSS signal. The frequency of the interference pattern was calculated with the Lomb-Scargle periodogram method ^11^. This algorithm calculates the spectral power for irregularly spaced time series. Reflections from elevation angles ranging between 2 deg and 30 deg were used. Satellite tracks, which contain less than 2.000 data points (equal to roughly 30 minutes of observation), were discarded.

The maximum peak in the spectral analysis of the GNSS signal strength was converted into the height of the GNSS antenna above the reflecting surface (Fig. S_1).

The reflector height over the DS lake level increases by 95 cm/y **(**Fig. S_2b). Adding to this the subsidence of the GNSS beach station of 15 cm/y, the DS lake level, derived from GNSS, drops by -110 cm/y (Fig. S_2c). The offsets in the time series due to the relocation of the GNSS station were corrected using height difference calculated from the average antenna height of the 7 days before and after the displacement. The reflection of the GNSS signal from land shows a constant reflector height **(**Fig. S_2d). This means the GNSS station / weather tower (Fig. 1) is not sinking locally but that the subsidence of the GNSS station is a larger scale phenomenon.

The reflection area of the GNSS signals corresponds in first order to an ellipse, which can be described by the first Fresnel zone

$a=\frac{b}{sinE};b=\sqrt{\frac{\lambda h}{sinE}+\left( \frac{\lambda}{2sinE} \right)^{2}}$ (2),

where *a* represents the semi-major axis and *b* the semi-minor axis, $\lambda$ the GNSS wavelength, *h* the height of the antenna phase center above the reflecting surface and *E* the satellite elevation angle ^12^.

For our GNSS antenna with a reflector height of 4.6 to 5.6 m the first Fresnel zone has a typical dimension of 50 x 4 m (mayor/minor axis) for a reflection angle “*E*” of 5 deg and 15 x 3 m for a reflection angle of 10 deg, respectively. Most of the signal comes from reflections between 5 deg and 10 deg. Hence, the area covered by the reflections of one satellite, is approx. 70 x 7 m. In this study, we combine the reflections of three satellites, which then cover an area, a “footprint”, of about 70 x 20 m.

| **E (deg.)** | **major axis (m)** | **minor axis (m)** |
| --- | --- | --- |
| 2 | 200 | 7 |
| 5 | 50 | 4 |
| 10 | 15 | 3 |
| 15 | 8 | 2 |

**Table 1:** Size of GNSS reflectometry footprint (ellipse) as function of reflection angle *E*.

**InSAR analysis**

We assess the surface displacement around the DS basin by InSAR analysis of 257 images acquired by Copernicus Sentinel-1 SAR sensor between 2014 and 2020. The dataset has a spatial resolution of 20 x 5 m and is acquired during descending passes in the period between October 2014 and April 2020. The temporal resolution of data is 12 days in the period from October 2014 to September 2016 and afterwards it improves to a 6 days repeat cycle. The SAR images are cropped to cover an area of 5 × 2 km centered on 31°25’56”N, 35°23’34”E and then they are co-registered to a common reference image.

For InSAR time series analysis, first, a network of Small Baseline (SB) interferograms ^13^ was formed. To preserve the interferometric coherence in the rapidly deforming areas, temporal baselines are kept short and each image is connected to the next two consecutive images. A digital elevation model from Shuttle Radar Topography Mission data with a spatial resolution of 90 × 90 m ^14^ was used to reduce the Earth’s reference and topographic phases from the interferograms.

In the next step of InSAR time series analysis, a temporal analysis of both amplitude and phase ^15,16^ is performed on the network of interferograms to identify the point candidates with high signal to noise ratio i.e. stable phase measurement in time. The candidates are selected in the first iteration such that the amplitude dispersion index is below a threshold value of 0.6. Then, the interferometric phase measurements of these candidates are statistically analyzed to improve the selection. The final number of points with stable phase measurement in time identified within the area of interest exceeds one hundred thousand.

Once InSAR points with high signal to noise ratio are identified, their interferometric phases are unwrapped and connected in time to estimate the time series of displacement. Finally, phase errors from atmospheric delays are reduced from the time series by spatial and temporal filtering.

The final InSAR measurements are in the satellite’s Line of Sight (LOS). Assuming the horizontal displacement is not significant, 1-D LOS measurement d_LOS_ is converted to vertical displacement, d_v_, by d_v_=d_LOS_/cos(theta) where theta~=43° is the incidence angle of the sensor in the study area.

The density and distribution of the InSAR points is high in Sentinel-1 results and the subsidence is reliably retrieved. The results in Figure S_4a show surface deformation in LOS near the Ein Gedi area with a distinct displacement along the shoreline, compare also to ^17^. To the Southwest of the Beach station, a maximum LOS displacement of about 18 cm/y away from the satellite is estimated from Sentinel-1. Assuming the horizontal displacement is not significant, the vertical displacement rate in this area is as high as 25 cm/y. The displacement rate at the closest InSAR point to SPA station is 1.5 cm/y in LOS away from the satellite with respect to the arbitrary InSAR reference point (Figure S_4a), equivalent to 2 cm/y of subsidence. The area near Beach station exhibits a displacement of approximately 7.4 cm/y in LOS away from the satellite, which corresponds to 10.1 cm/y subsidence.

The area close to Ein Gedi (SPA and Beach 1, 2, and 3 GNSS stations) are homogenously covered by InSAR measurement points making it possible to compare the InSAR time series of the surface displacement with GNSS data.

**Soil mechanics**

We consider here a simple 1D-soil compaction theory based on ^18, 19^ under the following assumptions: The Beach station is located on the clayey marl (lime-carbonates) deposits of the former Dead Sea lakebed. An unconfined, isotropic, homogeneous, fully saturated Dead Sea brine layer of marl of H = 20 m thickness is overlying a thick Holocene salt layer. The depth of this salt layer has been determined by various studies on the Western side of the Dead Sea ^e.g. 20^. The salt layer is mechanically stiffer than the marl ^21^ and therefore we consider only compaction of the Marl layer subject to be relevant for the immediate pore pressure changes. With incompressible fluid and soil particles, under small strains and Darcy’s law valid in all hydraulic gradients, we can use the 1D compaction theory to calculate primary consolidation based on a hydraulic head change of $\Delta h=1.1 m$ (the mean annual decline of the DS water level, see Fig. 4b)

$S_{p}=\frac{C_{c}*H}{1+e_{0}} \log\left( \frac{\sigma_{0}^{'}+ \Delta\sigma^{'}}{\sigma_{0}^{'}} \right)$ (3).

The following parameters are derived from laboratory soil consolidation tests ^22, 23^ for the Dead Sea lime carbonates: $e_{0}=1.0$, the initial void ratio, yielding a high porosity of n = 0.5 typical for marl sediments above the salt layer and $C_{c}\approx0.3-0.5$ as the compression index. With $\rho_{LC}=2750 kg/m^{3}$, the density of lime-carbonates and $\rho_{DS}=1240 kg/m^{3}$ the density of Dead Sea brine, the effective stress $\sigma_{0}^{'}=270 kPa$ and effective stress change $\Delta\sigma^{'}=13.38 kPa$ due to lowering of the water table can be calculated. This yields a primary consolidation of $S_{p}\approx6.3-10.5 cm$ for both limits of the compression index, respectively. To determine $t_{95}$, the time after which 95 % of the consolidation of the soil has happened, we use the time dependency formulation based on the solution of the pore pressure diffusion equation ^e.g. 24^

$t_{95}=\frac{{(H/N)}^{2}*T}{C_{v}}$ (4).

Here, for two drainage faces (N=2) and T = 1.129 the time factor ^19^ after 95% of consolidation, the coefficient of consolidation $C_{v}$ plays a crucial role. For the Dead Sea lime-carbonates this value has shown a range over more than 3 orders of magnitude ^22^ depending on the Atterberg limits of solid plasticity and liquid limit. We here assume values of $C_{v}=1.0- 3.0$. With these parameters, the time needed for 95% primary consolidation is $t_{95}=1,19-3.58 yr$. For a high coefficient of consolidation (*C_v_* = 3.0) we therefore receive a primary consolidation time of roughly 14 months for the 20 m thick marl layer.

Also, secondary consolidation occurs due to plasticity and creep of the clay. Several parameters need to be defined for the following equation

$S_{s}=\frac{H}{1+e_{0}} C_{\alpha}\log\left( \frac{t}{t_{95}} \right)$ (5).

$C_{\alpha}$ is the secondary compression index and is usually estimated by the change of void ratio over time. It can be assumed to be time independent for low $t/t_{95}$ ratios as common in field measurements. Especially, without vertical drainage and when the final effective stress is in the range of the primary effective stress, $C_{\alpha}/C_{C}$ is very low and secondary consolidation is insignificant ^19^. However, for our problem this is unlikely as for inorganic clays and silt $C_{\alpha}=0.05*C_{C}$, and thus $C_{\alpha}$ is in the range of $0.015-0.025$. Hence, the magnitude after an additional year of compaction $(t=t_{95}+12 month)$ lies between $S_{s}\approx3.9$ and $6.5 cm$ respectively. As it lies in the same order as the primary consolidation, secondary consolidation is not negligible for the Dead Sea mud.

Given the ongoing decline of the Dead Sea lake level, we assume that primary and secondary consolidation occur continuously at this location and, thus, we simply sum both solutions and receive a range of lime-carbonate soil compaction.

The delay between land displacements at the Beach site and the seasonal fluctuation of the DS level (Fig. 4a) can be explained by considering the influence of the latter on the groundwater pressure distribution.

The description the water table motion in a beach is a classical hydrogeologic problem. Specifically, the residual sea level fluctuation can be viewed as a long-term tide characterized by an amplitude $\zeta_{0}=15$ cm and a period $t_{0}=365$ days.

A preliminary analysis can be carried out through a number of analytical solutions proposed for simplified geometry, geological setting, and tidal signal ^21,25^. In the simplest condition, which suffices in the context of the present work to explain the observations, let’s consider a homogeneous beach characterized by an isotropic permeability $K$ and effective porosity $n_{e}$. The groundwater flow is assumed essentially horizontal so that the pressure distribution is hydrostatic (Dupuit’s assumption). Therefore, the governing equation for the water table height $\zeta$ above the mean sea level is (Bouissinesq’s equation):

$\frac{\partial\zeta}{\partial t}=\frac{K}{n}\frac{\partial}{\partial x}\left( \zeta\frac{\partial\zeta}{\partial x} \right)$ (6).

With an almost vertical beach, a sea level fluctuation characterized by a simple harmonic motion $\zeta=\zeta_{0}\sin\omega t$with a tidal period $t_{0}={2\pi}/\omega$ and a small tidal amplitude (i.e., $\zeta_{0}$much smaller than the aquifer thickness), and imposing $\zeta=0$ at an infinite distance $x$ from the shoreline, the solution of the above equation reads^:^

$\zeta\left( x,t \right)=\zeta_{0}\sin\left( \omega t-kx \right)e^{-kx}$ (7).

where the wave number $k=\sqrt{\frac{n_{e}\omega}{2K}}$. The wave amplitude $h_{max}$ and the time lag $t_{L}$ of the water table oscillation at a distance $x$ from the shoreline are:

$$\zeta_{max}=\zeta_{0}e^{-kx}$$

$t_{L}=x\sqrt{\frac{t_{0}n_{e}}{4\pi K}}$ (8).

We consider that the clayey marl deposits of the former Dead Sea lakebed is characterized by $n_{e}=0.5n=0.25$ and $K={10}^{-5}$ m/s because of the fast dissolution and erosion that typically affect the deposits. The shortest distance $x$ of the Beach station from the shoreline changed over time because of the DS level lowering. It ranged approx. from 10 to 35 m. Introducing these values in the previous equation yields $t_{L}=29$ days and $t_{L}=101$ days, respectively.

Notice that $\zeta_{max}=9.0$cm at x=10 m and $\zeta_{max}=2.6$cm at x= 35 m distance, i.e. 60% and 17% of $\zeta_{0}$, respectively.

**Figures** - **Electronic supplement:**


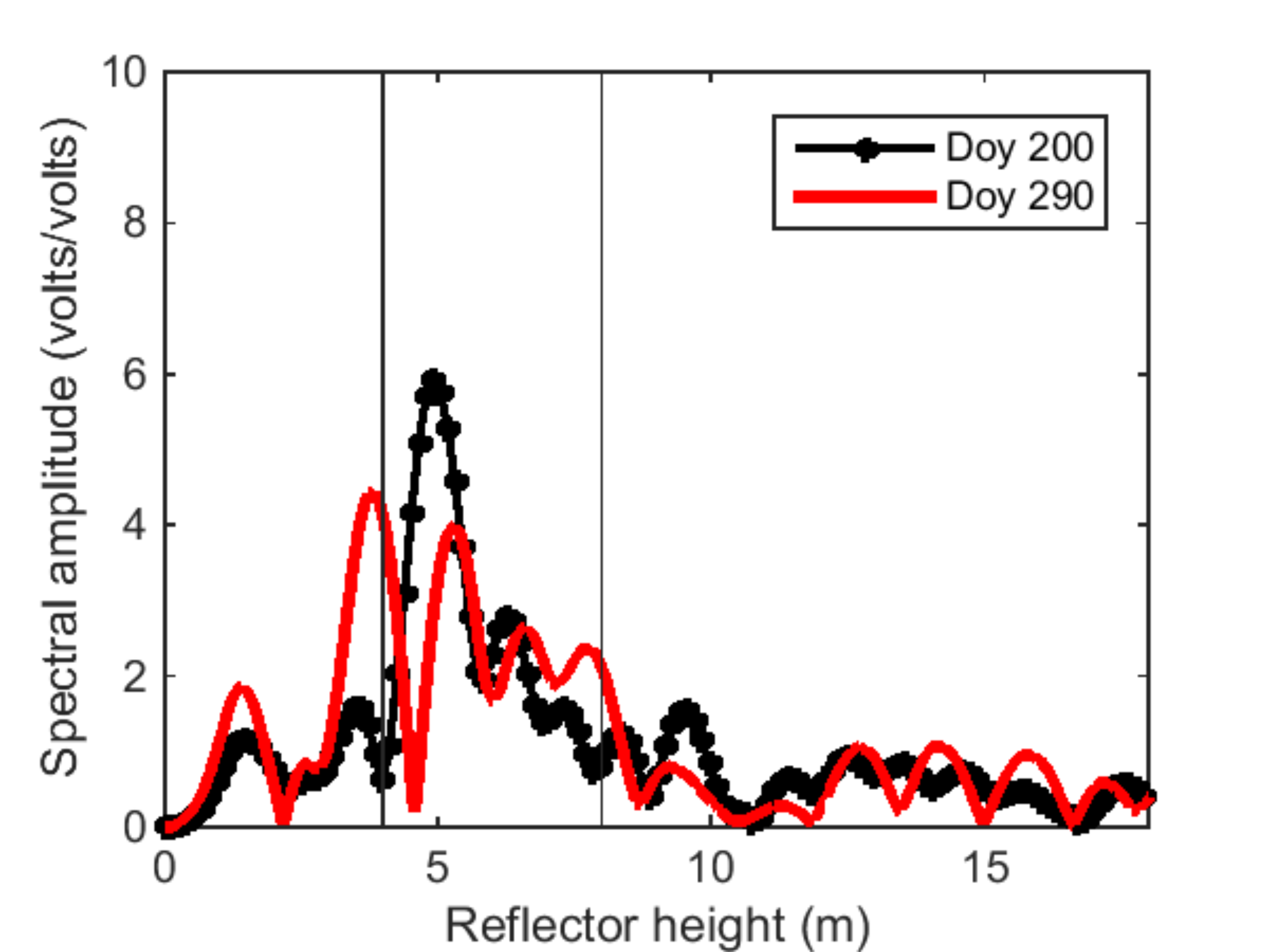


**Fig. S_1:**

Lomb-Scargle periodogram from the Signal-To-Noise Ratio of the GPS satellite #26. On day 200 (black line) the maximum peak at 4.9m corresponds to the reflector height over the Dead Sea. On day 290 (red line) the standard algorithm would detect the reflector height over the lake at the maximum peak of 3.8m. However, due to the strong horizontal retreat of the DS, this peak corresponds to the reflections from land. The second largest peak at 5.2 m characterizes the reflections from the DS. Our new algorithm uses a window function for the maximum peak detection shown by the vertical lines.


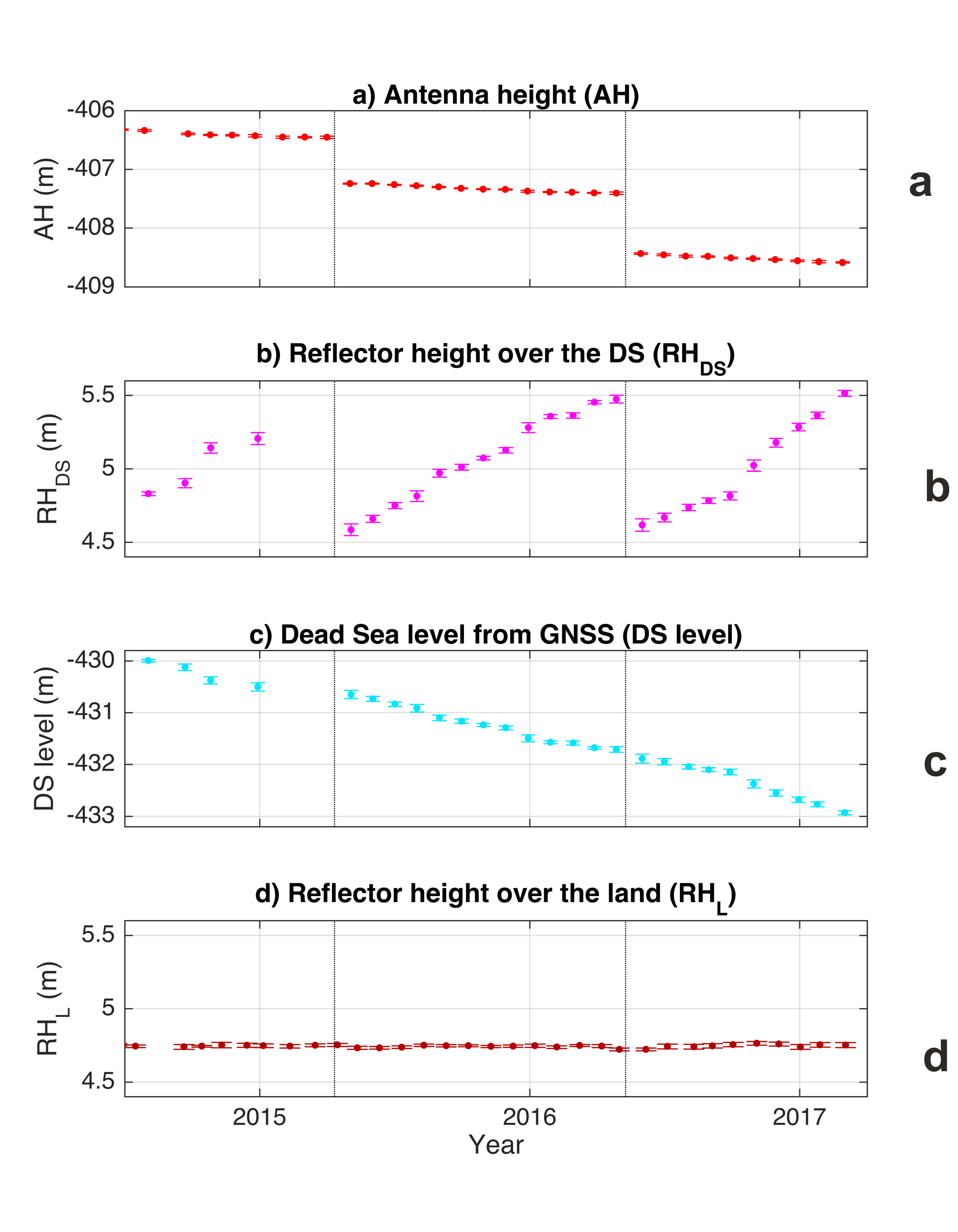


**Fig. S_2:**

**(a)** Changes in antenna height from GNSS (red). The station was moved closer to the lake on 10^th^ of April 2015 and again on 9^th^ of May 2016 (dotted vertical lines). The small data gap in 2014 is due to missing observations. **(b)** Reflector height over water (magenta) is increasing with time corresponding to the drop of the lake level. The gap in the time series is because the water moved to far away from the station for a reliable determination. **(c)** DS lake level (blue) derived from the difference of GNSS antenna height (corrected for the offsets due to the displacements) and the reflector height over water. **(d)** Reflector height over land (brown). This shows that the GNSS station is stable and not sinking into the ground. Error bars in all subplots indicate the standard deviation of the monthly averages based on daily values.


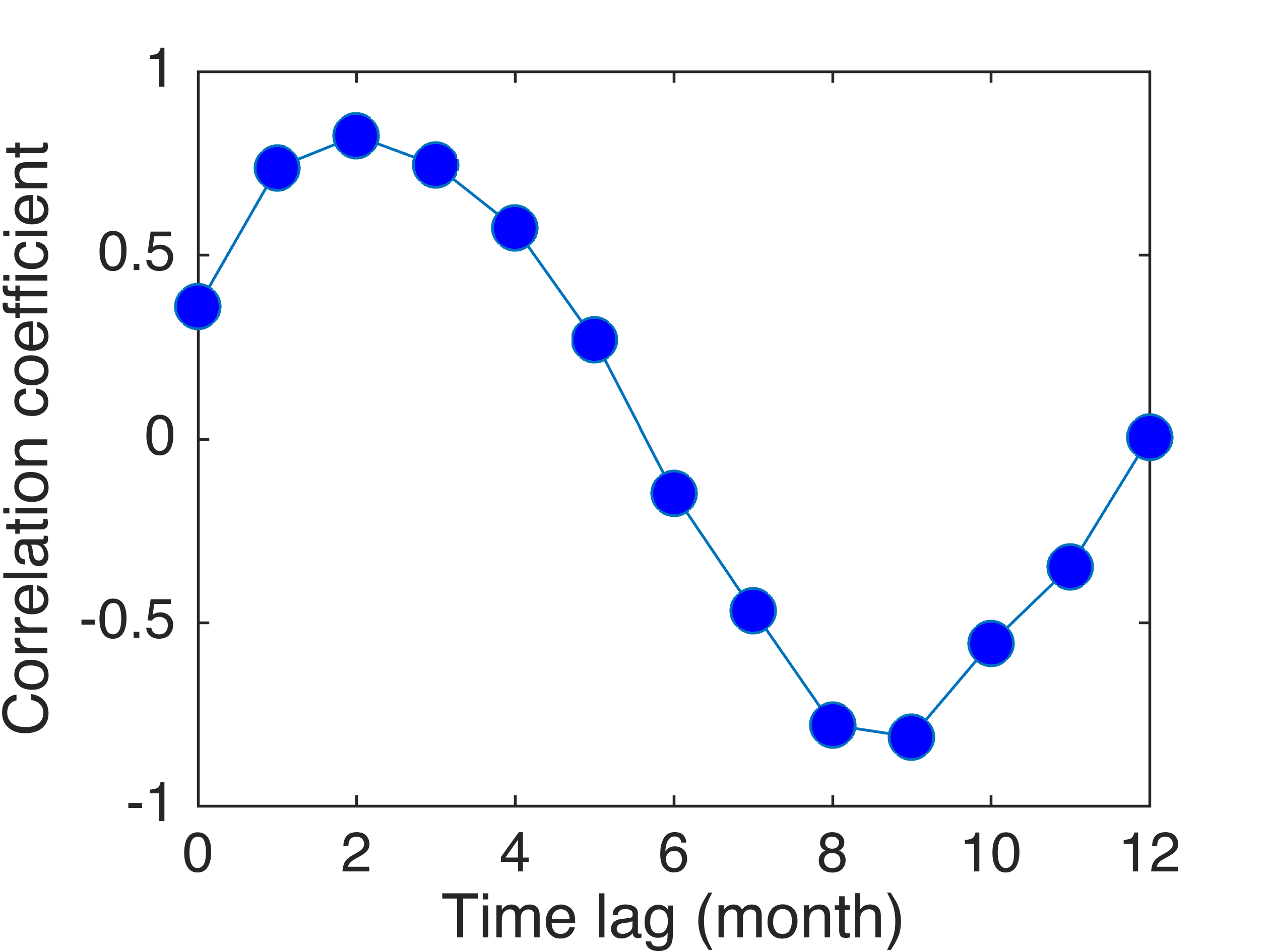


**Fig. S_3:**

Correlation between the time series of the DS lake level and the subsidence at Beach stations on a monthly basis for different time delays at the Beach station(s). The maximum value of 0.84 is reached for a time shift of 2 months.

**
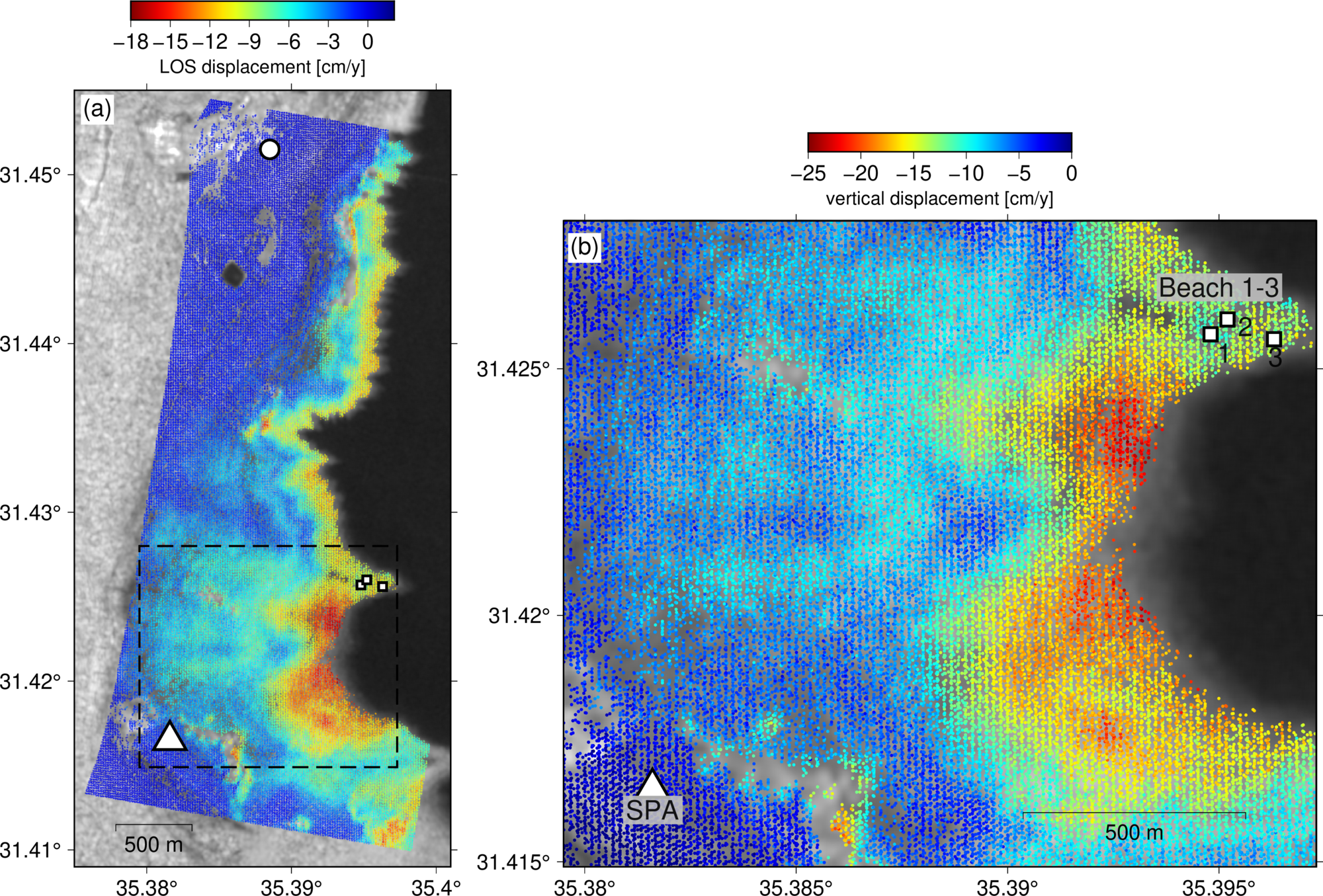
**

**Fig. S_4:**

InSAR-based maps of surface displacement rate obtained from SBAS time series analysis of 2014-2020 data from [Copernicus](http://www.esa.int/Our_Activities/Observing_the_Earth/Copernicus) Sentinel-1 © ESA overlaid on average SAR amplitude image **(a)** Line of Sight rate of displacement. Dashed line indicates the close up view displayed in (b). **(b)** vertical displacement rate in a close up view for the region near Ein Gedi. The vertical displacement values are estimated assuming horizontal displacements are negligible. The location of the SPA GNSS station is displayed by a white triangle. Beach 1-3 stations are indicated by white squares. The white circle represents the arbitrary spatial reference point for InSAR.

**Fig. S_5c:**

Background image (DigitalGlobe) from 14^th^ August.2013 with a resolution of 50 cm. Shorelines are extracted from 10 m resolution Sentinel 2 (2016) and 3 m resolution Rapideye (2017) images, respectively.


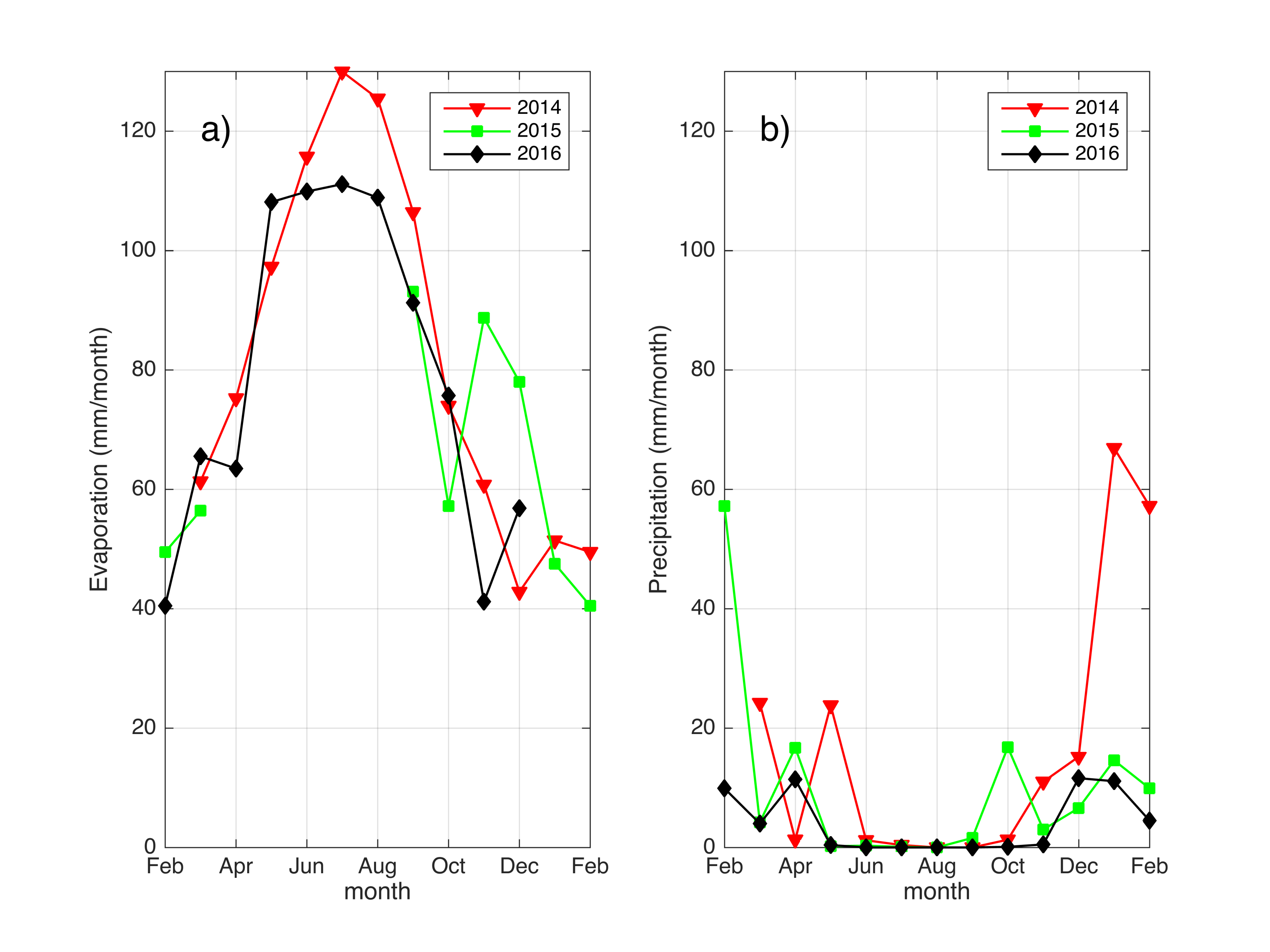


**Fig. S_6:**

**(a)** Monthly sum of evaporation at the Beach station (Fig. 1) derived from ^26^, our basis for the cumulative values in Fig. 4b. **(b)** Monthly sum of precipitation observed at the Beach station. Strong precipitation in winter 2014/15 produces a small increase in the lake level of the DS (Fig. 4b, blue triangles).

**References**

1. Ge, M., Gendt, G., Dick, G., & Zhang, F. Improving the carrier phase ambiguity resolution in global GPS network solution. *Journal of Geodesy*, 79, 103-110, DOI: 20.2007/s00290-005-0447-0 (2005).

2. Ge, M., Gendt, G., Rothacher, M., Shi, C., & Liu, J. Resolution of GPS Carrier Phase Ambiguities in Precise Point Positioning (PPP) with Daily Observations, *Journal of Geodesy*, 82(7), 389-399, DOI:10.1007/s00190-007-0187-4 (2008).

3. Petit, G. & Luzum, B. (eds.) IERS Conventions 2010, *IERS Technical Note*; 36, Frankfurt am Main: Verlag des Bundesamts für Kartographie und Geodäsie (2010).

4. Zumberge, J. F., Heflin, M. B., Jefferson, D. C., Watkins, M. M., & Webb, F. H. Precise point positioning for the efficient and robust analysis of GPS data from large networks, *Journal of Geophysical Research*, 102, B3, 5005-5017 (1997).

5. Santamaría-Gómez, A. & C. Watson, C. Remote leveling of tide gauges using GNSS reflectometry: Case study at Spring Bay, Australia. *GPS Solutions*, 21(2), 451-459. [https://doi.org/10.1007/s10291-016-0537-x](https://doi.org/10.1007%2Fs10291-016-0537-x) (2017).

6. Larson, K. M., Ray, R. D. & S.P. Williams, S. P. A ten year comparison of water levels measured with a geodetic GPS receiver versus a conventional tide gauge. *J. Atmos. Ocean Tech,* Vol. 34(2), 295-307. doi: 10.1175/JTECH-D-16-0101.1 (2017).

7. Frydman, S., Charrach, J. & Goretsky, I. A geotechnical study of evaporitic, lacustrine sediments in the saline environment of the Dead Sea area. *Engineering Geology*, 181, 309-322, doi:10.1016/j.enggeo.2014.08.028 (2014).

8. Strandberg, J. T., Hobiger, T. & Haas, R. Improving GNSS-R sea level determination through inverse modeling of SNR data. *Radio Sci*., 51, 1286–1296. [https://doi.org/10.1002/2016RS006057](https://doi.org/10.1002%2F2016RS006057) (2016).

9. Gurtner, W. & Estey, L. RINEX: The receiver independent exchange format version 2.11. http://igscb.jpl.nasa.gov/igscb/ data/format/rinex211.txt (2007).

10. Larson, K., Small, E., Gutmann, E., Bilich, A., Braun, J. & Zavorotny, V. Use of GPS receivers as a soil moisture network for water cycle studies. *Geophysical Research Letters*, 35(24), L24 405. doi:10.1029/2008GL036013 (2008).

11. Press, W. H. & Rybicki, G. B. Fast algorithm for spectral analysis of unevenly spaced data. *Astrophysical Journal*, 338, 277–280. doi:10.1086/167197 (1989).

12. Larson, K.M. & Nievinski, F.-G. GPS snow sensing: results from the EarthScope Plate Boundary Observatory. *GPS Solution*, 17, 41–52. doi:10.1007/s10 291–012–0259–7 (2012).

13. Berardino, P., et al. A new algorithm for surface deformation monitoring based on small baseline differential SAR interferograms. *IEEE Transactions on geoscience and remote sensing*, 40.11, 2375-2383 (2002).

14. Farr, T. G. & Kobrick, M., Shuttle Radar Topography Mission produces a wealth of data. *Eos, Transactions American Geophysical Union,* 81.48, 583-585 (2000).

15. Hooper, A., A multi‐temporal InSAR method incorporating both persistent scatterer and small baseline approaches. *Geophysical Research Letters,* 35.16 (2008).

16. Hooper, A., Segall, P. & Howard Zebker, H. Persistent scatterer interferometric synthetic aperture radar for crustal deformation analysis, with application to Volcán Alcedo, Galápagos." *Journal of Geophysical Research: Solid Earth* 112.B7 (2007).

17. Nof, R. N., M. Abelson, E. Raz, Y. Magen, S. Atzori, S. Salvi & Baer, G. SAR interferometry for sinkhole early warning and susceptibility assessment along the Dead Sea, Israel, *Remote Sens.*, *11*(1), 89, doi:10.3390/rs11010089 (2019).

18. Terzaghi, K., *Theoretical Soil Mechanics*, John Wiley & Sons, New York (1943).

19*.* Terzaghi, K., Peck, R.B. & Mesri, G. *Soil mechanics in engineering practice*, John Wiley & Sons (1996).

20. Shalev, E. & Lyakhovsky, V. Viscoelastic damage modeling of sinkhole formation, *J. Struct. Geol.*, *42*, 163–170, doi:10.1016/j.jsg.2012.05.010 (2012).

21. Carr, P. A. & Van der Kamp, G. S., Determining aquifer characteristics by the tidal method, *Water Resources Research*, 5(5), 1023-1031 (1969).

22. Ezersky, M. G. & Frumkin, A. Fault - Dissolution front relations and the Dead Sea sinkhole problem, *Geomorphology*, *201*, 35–44, doi:10.1016/j.geomorph.2013.06.002 (2013).

23. Ismeik, M., Ashteyat, A.M. & Ramadan, K.Z. Stabilisation of fine-grained soils with saline water, *Eur. J. Environ. Civ. Eng.*, *17*(1), 32–45, doi:10.1080/19648189.2012.720399 (2013).

24. Verruijt, A. Soil Mechanics. Delft University of Technology (2012).

25. Nielsen, P., Tidal dynamics of the water table beaches, *Water Resources Research*, 26(9), 2127-2134 (1990).

26. Gräbe, A., T. Rödiger, K. Rink, T. Fischer, F. Sun, W. Wang, C. Siebert & Kolditz, O. Numerical analysis of the groundwater regime in the western Dead Sea escaprment, Israel + West Bank, *Environ. Earth Sci.*, *69*, 571–585, doi:10.1007/s12665-012-1795-8 (2013).
